# Pasireotide for acromegaly: long-term outcomes from an extension to the Phase III PAOLA study

**DOI:** 10.1530/EJE-19-0762

**Published:** 2020-03-27

**Authors:** Annamaria Colao, Marcello D Bronstein, Thierry Brue, Laura De Marinis, Maria Fleseriu, Mirtha Guitelman, Gerald Raverot, Ilan Shimon, Jürgen Fleck, Pritam Gupta, Alberto M Pedroncelli, Mônica R Gadelha

**Affiliations:** 1 Università Federico II di Napoli, Naples, Italy; 2 University of São Paulo Medical School, São Paulo, Brazil; 3 Aix-Marseille Université, Institut National de la Santé et de la Recherche Médicale INSERM U1251, Marseille Medical Genetics and Assistance Publique Hôpitaux de Marseille (APHM), Hôpital de la Conception, Marseille, France; 4 Università Cattolica del Sacro Cuore, Rome, Italy; 5 Northwest Pituitary Center, Oregon Health & Science University, Portland, Oregon, USA; 6 Endocrinology Division, Carlos G Durand Hospital, Buenos Aires, Argentina; 7 Groupement Hospitalier Est, Hospices Civils de Lyon and Lyon 1 University, Lyon, France; 8 Rabin Medical Center and Sackler School of Medicine, Tel-Aviv University, Petah-Tiqva, Israel; 9 Novartis Pharma AG, Basel, Switzerland; 10 Novartis Healthcare Private Limited, Hyderabad, India; 11 Hospital Universitário Clementino Fraga Filho, Universidade Federal do Rio de Janeiro, Rio de Janeiro, Brazil

## Abstract

**Objective:**

In the Phase III PAOLA study (clinicaltrials.gov: NCT01137682), enrolled patients had uncontrolled acromegaly despite ≥6 months of octreotide/lanreotide treatment before study start. More patients achieved biochemical control with long-acting pasireotide versus continued treatment with octreotide/lanreotide (active control) at month 6. The current work assessed the extent of comorbidities at baseline and outcomes during a long-term extension.

**Design/methods:**

Patients receiving pasireotide 40 or 60 mg at core study end could continue on the same dose in an extension phase if biochemically controlled or receive pasireotide 60 mg if uncontrolled. Uncontrolled patients on active control were switched to pasireotide 40 mg, with the dose increased at week 16 of the extension if still uncontrolled (crossover group). Efficacy and safety are reported to 304 weeks (~5.8 years) for patients randomized to pasireotide (core + extension), and 268 weeks for patients in the crossover group (extension only).

**Results:**

Almost half (49.5%; 98/198) of patients had ≥3 comorbidities at core baseline. During the extension, 173 patients received pasireotide. Pasireotide effectively and consistently reduced GH and IGF-I levels for up to 5.8 years’ treatment; 37.0% of patients achieved GH <1.0 µg/L and normal IGF-I at some point during the core or extension. Improvements were observed in key symptoms. The long-term safety profile was similar to that in the core study; 23/173 patients discontinued treatment because of adverse events.

**Conclusions:**

In this patient population with a high burden of comorbid illness, pasireotide was well tolerated and efficacious, providing prolonged maintenance of biochemical control and improving symptoms.

## Introduction

Acromegaly is an endocrine disorder most commonly caused by chronic excess secretion of growth hormone (GH) from a pituitary adenoma and subsequent hepatic hypersecretion of insulin-like growth factor 1 (IGF-I) (1). Chronic hypersecretion of GH and IGF-I results in multiple comorbidities, particularly cardiometabolic complications (2), that not only increase mortality risk but also imposes a substantial burden of illness on patients. Achieving biochemical control in line with guideline recommendations (3) can reduce morbidity and may mitigate the increased mortality risk associated with acromegaly (4, 5).

First-generation somatostatin analogues (long-acting octreotide and lanreotide) are the current first-line standard of medical care in patients with acromegaly (3). Despite the clinical success of these agents (6), many patients remain uncontrolled (7, 8) and therefore exposed to the deleterious health consequences associated with elevated GH and IGF-I levels. Pasireotide is a multireceptor-targeted second-generation somatostatin analogue that has demonstrated superior efficacy over first-generation somatostatin analogues in two randomized, prospective, Phase III clinical studies (9, 10). In study C2305, conducted in patients with active acromegaly who had not received medical therapy at study entry, biochemical control (defined as GH <2.5 µg/L and normal IGF-I) was achieved by a significantly greater proportion of patients receiving long-acting pasireotide than long-acting octreotide (31.3 vs 19.2%; *P* = 0.007) at month 12 (9). The PAOLA study (C2402) evaluated patients with uncontrolled acromegaly while on maximal approved doses of long-acting octreotide or lanreotide prior to the start of the study (10). Significantly more patients who were treated with long-acting pasireotide achieved biochemical control (GH <2.5 µg/L and normal IGF-I) than patients who continued treatment with long-acting octreotide or lanreotide at month 6 (15.4% (long-acting pasireotide 40 mg) and 20.0% (long-acting pasireotide 60 mg) vs 0%; *P* = 0.0006 and *P* < 0.0001, respectively) (10).

The current work includes a detailed analysis of the baseline comorbidities of patients who participated in the PAOLA study, along with efficacy and safety findings for up to 5.8 years of follow-up. Notably, biochemical control in the core phase of the PAOLA study was defined according to accepted treatment guidelines at the time of study start (11). However, updated treatment guidelines have now been published recommending a more stringent definition of biochemical control (GH <1.0 μg/L and normalized IGF-I) (3). To ensure the clinical relevance of the data reported here, biochemical control in the extension phase of the PAOLA study is defined according to these up-to-date treatment guidelines.

## Methods

### Eligibility criteria

Patient eligibility criteria for the core study have been reported previously (10). Briefly, male and female patients aged ≥18 years with uncontrolled acromegaly, defined as mean GH >2.5 μg/L *and* IGF-I >1.3 times the sex- and age-adjusted upper limit of normal (ULN), were enrolled. Patients had received treatment with long-acting octreotide 30 mg or lanreotide 120 mg/28 days for ≥6 months prior to screening. Patients could have had ≥1 prior pituitary surgery and could have received combination therapy with a dopamine agonist or GH-receptor antagonist.

The study was conducted in accordance with the Declaration of Helsinki, and an independent ethics committee or institutional review board for each study site approved the study protocol (see Supplementary Appendix for full details, see section on supplementary materials given at the end of this article). All patients provided written informed consent to participate in the study.

### Study design

This was an extension to a prospective, multicentre, randomized, parallel-group, 6-month core study. In the core study, patients were randomized to double-blind long-acting pasireotide 40 mg/28 days or long-acting pasireotide 60 mg/28 days, or continued treatment with open-label long-acting octreotide 30 mg or lanreotide 120 mg every 28 days (10). No dose increases were permitted during the core phase. All patients who completed the core study were eligible to participate in the extension phase. The last visit of the core study was considered as the first visit of the extension phase. The phase between the end of the core study and the second visit of the extension phase was defined as the ‘bridging phase’, during which patients continued to receive their randomized treatment. After this time, patients who were receiving pasireotide 40 or 60 mg and who had GH <2.5 µg/L and normalized IGF-I at the end of the core study continued to receive the same dose of double-blind pasireotide during the extension. Patients who were uncontrolled at week 24 of the core study with pasireotide 40 or 60 mg could receive open-label pasireotide 60 mg. Patients in the active control group who were uncontrolled at week 24 of the core study were switched to open-label pasireotide 40 mg (referred to hereafter as the crossover group); a dose increase to pasireotide 60 mg was permitted at week 16 of the extension phase if GH <2.5 µg/L and normalized IGF-I was not achieved. Dose decreases of 20 mg were permitted for tolerability issues in patients randomized to receive pasireotide. If a patient in the crossover group experienced a severe drug-related adverse event (AE), dose decreases to the next-available lower dose were permitted. Previous dosing was resumed once the tolerability issue had resolved.

The principal investigator was responsible for educating the patient on the signs and symptoms of hyperglycaemia. It was recommended that established guidelines by expert international diabetes associations, such as the American Diabetes Association and European Association for the Study of Diabetes, were followed for the management of any instances of hyperglycaemia during the study. Any patients in whom fasting plasma glucose (FPG) levels were >130 mg/dL, or 2-h post-prandial capillary glucose was ≥180 mg/dL on two consecutive measurements that were ~14 days apart, and/or glycated haemoglobin (HbA_1c_) was >7% were to be evaluated by a diabetes specialist for appropriate treatment.

### Assessments

Assessments reported here include the following: proportion of patients with biochemical control (GH <1.0 μg/L and normalized IGF-I); mean GH and IGF-I levels over time; number of patients who had a dose increase/decrease in the extension phase; changes in signs and symptoms; baseline characteristics of early and late responders during the extension phase; and safety. Additionally, baseline demographics according to comorbidity group were determined. Patients were classified into five groups of comorbidities commonly associated with acromegaly: glucose-related, endocrine-related, lipid-related, vascular, and all other acromegaly-related disorders (see Supplementary Appendix for definitions). Baseline demographics by comorbidity group were determined for the total population. Details on the GH and IGF-I assays used have been published previously (10).

### Definitions of diabetic status

- Diabetic was defined as patients taking antidiabetic medication, or with prior history of diabetes mellitus, or with HbA_1c_ ≥6.5% (≥47.5 mmol/mol) or FPG ≥126 mg/dL (≥7.0 mmol/L).
- Pre-diabetic was defined as patients not qualifying as diabetic and with HbA_1c_ ≥5.7% (≥38.8 mmol/mol) and <6.5% (<47.5 mmol/mol) or FPG ≥100 (≥5.6 mmol/L) and <126 mg/dL (<7.0 mmol/L).
- Normal glucose tolerance was defined as patients not qualifying as diabetic or pre-diabetic and with HbA_1c_ <5.7% (<38.8 mmol/mol) and/or FPG <100 mg/dL (<5.6 mmol/L).

### Statistical analyses

Treatment groups were based on randomized treatment at core baseline. Efficacy and safety are reported up to study end: 303.9 weeks (~5.8 years) after start of treatment (core + extension) for patients randomized to receive pasireotide, and 268.0 weeks for the crossover group (extension only). Baseline conditions and patient demographics, as well as efficacy and safety data, are summarized descriptively.

## Results

### Core study baseline conditions and patient demographics

One hundred and ninety-eight patients entered the core study. Individual disease conditions, as reported by the investigator, comprising each comorbidity group at core baseline (*n* = 198) are shown in Table 1. Patient baseline characteristics were similar across the comorbidity groups, with glucose- and endocrine-related comorbidities the most common in the overall patient population (Table 2); 49.5% (98/198) of patients had comorbidities in three or more comorbidity groups (Table 3 and Supplementary Fig. 1). Of the 198 patients in the core study, 67.2% (132/198) were defined according to predefined study criteria as diabetic, 20.7% (41/198) as pre-diabetic, and 11.6% (23/198) as having normal glucose tolerance. Of the 145 patients with investigator-reported baseline glucose-related disorders, 71.7% were defined as diabetic at baseline, while 28.3% were pre-diabetic at baseline (Table 2).

**Table 1:** Most common conditions within each comorbidity group classification reported by the investigator at core baseline (occurring in ≥5% of patients in any treatment group). Comorbidities listed as reported by the investigator. Data based on preferred terms from patient medical history. Complete list of comorbidities for each comorbidity group classification is provided in the Supplementary Appendix.

Comorbidity, *n* (%)	Long-acting pasireotide 40 mg, *n* = 65	Long-acting pasireotide 60 mg, *n* = 65	Active control, *n* = 68
Vascular disorders			
Hypertension	17 (26.2)	27 (41.5)	36 (52.9)
Glucose-related disorders			
Diabetes mellitus	18 (27.7)	11 (16.9)	16 (23.5)
Impaired glucose tolerance	10 (15.4)	10 (15.4)	11 (16.2)
Type 2 diabetes mellitus	6 (9.2)	9 (13.8)	4 (5.9)
Lipid-related disorders			
Dyslipidaemia	10 (15.4)	15 (23.1)	8 (11.8)
Hypercholesterolaemia	6 (9.2)	5 (7.7)	5 (7.4)
Endocrine-related disorders			
Goitre	9 (13.8)	14 (21.5)	23 (33.8)
Hypothyroidism	10 (15.4)	10 (15.4)	12 (17.6)
Adrenal insufficiency	8 (12.3)	6 (9.2)	10 (14.7)
Hypopituitarism	7 (10.8)	4 (6.2)	5 (7.4)
Hyperprolactinaemia	5 (7.7)	5 (7.7)	3 (4.4)
Hypogonadism	4 (6.2)	6 (9.2)	5 (7.4)
Diabetes insipidus	4 (6.2)	4 (6.2)	2 (2.9)
Secondary hypothyroidism	6 (9.2)	4 (6.2)	4 (5.9)
Secondary hypogonadism	5 (7.7)	5 (7.7)	6 (8.8)
Secondary adrenocortical insufficiency	1 (1.5)	5 (7.7)	3 (4.4)
Other acromegaly-related disorders			
Depression	7 (10.8)	2 (3.1)	4 (5.9)
Headache	4 (6.2)	3 (4.6)	1 (1.5)
Osteoarthritis	4 (6.2)	3 (4.6)	6 (8.8)
Carpal tunnel syndrome	5 (7.7)	1 (1.5)	5 (7.4)
Carpal tunnel decompression	3 (4.6)	2 (3.1)	4 (5.9)
Insomnia	2 (3.1)	4 (6.2)	1 (1.5)
Osteoporosis	3 (4.6)	2 (3.1)	4 (5.9)
Aortic valve incompetence	0 (0.0)	1 (1.5)	4 (5.9)
Haemangioma of the liver	0 (0.0)	0 (0.0)	4 (5.9)

**Table 2:** Core study baseline demographics by comorbidity group. Data based on 198 patients in the core baseline population.

	Glucose-related disorders	Endocrine-related disorders	Vascular disorders	Lipid-related disorders	All other acromegaly-related disorders
*n*	145	127	80	56	111
Mean age, years (s.d.)	47.1 (14.0)	45.8 (13.7)	51.9 (12.2)	52.3 (13.6)	48.6 (12.6)
Gender, *n* (%)					
Male Female	59 (40.7) 86 (59.3)	56 (44.1) 71 (55.9)	35 (43.8) 45 (56.3)	17 (30.4) 39 (69.6)	41 (36.9) 70 (63.1)
Mean weight, kg (s.d.)	85.4 (18.9)	86.6 (19.9)	89.9 (20.1)	85.6 (19.3)	85.8 (20.9)
Mean BMI, kg/m^2^ (s.d.)	29.9 (6.0)	29.8 (5.9)	31.5 (6.2)	31.1 (5.7)	29.9 (6.2)
Baseline diabetic status,* *n* (%)					
Diabetic Pre-diabetic Normal glucose tolerance Missing	104 (71.7) 41 (28.3) 0 (0.0) 0 (0.0)	94 (74.0) 20 (15.7) 13 (10.2) 0 (0.0)	64 (80.0) 14 (17.5) 1 (1.3) 1 (1.3)	46 (82.1) 7 (12.5) 3 (5.4) 0 (0.0)	81 (73.0) 21 (18.9) 9 (8.1) 0 (0.0)
Mean baseline GH, µg/L (s.d.)	12.7 (23.8)	12.4 (22.9)	10.3 (13.9)	9.0 (15.8)	10.6 (20.7)
Mean baseline IGF-I, x ULN (s.d.)	2.9 (1.1)	2.7 (1.1)	3.1 (1.1)	3.0 (1.1)	2.9 (1.1)

*See Methods section for definitions of diabetic status.

**Table 3:** Number of patients from the randomized population (*n* = 198) who had one or more comorbidities at baseline.

Number of comorbidities at baseline	Proportion of randomized population, *n* (%)
1	42 (21.2)
2	41 (20.7)
3	51 (25.8)
4	34 (17.2)
5	13 (6.6)

### Patient disposition: core phase

In the pasireotide 40 mg, pasireotide 60 mg and crossover groups, respectively, 59 (90.8%), 57 (87.7%) and 65 (95.6%) patients completed the 24-week core study (Supplementary Fig. 1); reasons for discontinuation included AEs (*n* = 2, 4 and 0), consent withdrawal (*n* = 2, 2 and 2), administrative problems (*n* = 2, 1 and 0) and protocol deviations (*n* = 0, 1 and 1).

### Patient disposition: extension phase

Of the 174 patients who entered the extension phase, 173 were treated: pasireotide 40 mg, *n* = 57; pasireotide 60 mg, *n* = 54; crossover, *n* = 62. Overall, 65.9% (114/173) were defined as diabetic, 23.7% (41/173) as pre-diabetic, and 10.4% (18/173) as having normal glucose tolerance at baseline. In total, 28 (49.1%), 25 (46.3%), and 34 (54.8%) patients in the pasireotide 40 mg, 60 mg, and crossover groups, respectively, completed the extension phase of the study (Supplementary Fig. 1). Discontinuations during the extension phase were as follows: 29 patients from the pasireotide 40 mg group (unsatisfactory effect, *n* = 15; withdrawal of consent, *n* = 6; AE, *n* = 4; death, *n* = 2; protocol deviation, *n* = 2), 29 patients from the pasireotide 60 mg group (unsatisfactory effect, *n* = 9; withdrawal of consent, *n* = 8; AE, *n* = 8; administrative issues, *n* = 2; loss to follow-up, *n* = 1; protocol deviation, *n* = 1), and 28 patients from the crossover group (unsatisfactory effect, *n* = 13; withdrawal of consent, *n* = 8; AE, *n* = 7). Median (range) duration of pasireotide exposure from start of treatment to end of study was 152.1 (11.9–303.9) weeks in the pasireotide 40 mg group, 149.6 (4.0–295.4) weeks in the pasireotide 60 mg group, and 201.6 (16.0–268.0) weeks in the crossover group.

### Long-term efficacy: biochemical response

In all three treatment groups, mean GH and IGF-I levels were consistently suppressed throughout the duration of the extension phase until study end (Table 3). Biochemical response rates (GH <1.0 µg/L and normal IGF-I) varied throughout the extension: 1.8–10.5% and 3.7–20.4% for patients randomized to pasireotide 40 mg and 60 mg, respectively, and 1.6–11.3% for patients in the crossover group (Table 4).

**Table 4:** Response rates (GH <1.0 µg/L and normal IGF-I) during the extension phase.

Study visit*	Pasireotide 40 mg, *n* = 57			Pasireotide 60 mg, *n* = 54			Crossover group, *n* = 62		
	Response rate,^‡^ *n* (% ITT) (% ongoing pts)	Mean GH, µg/L (s.d.)	sIGF-I, (s.d.)	Response rate,^‡^ *n* (% ITT) (% ongoing pts)	Mean GH, µg/L (s.d.)	sIGF-I, s.d.	Response rate,^‡^ *n* (% ITT) (% ongoing pts)	Mean GH, µg/L (s. d.)	sIGF-I, (s.d.)
Baseline^†^	–	13.5 (28.2)	2.5 (1.0)	–	11.8 (20.7)	2.7 (1.0)	–	15.4 (67.0)	2.6 (1.0)
Week 52	5 (8.8) (10.4)	4.0 (6.7)	1.6 (1.2)	9 (16.7) (19.6)	3.6 (6.2)	1.3 (0.9)	5 (8.1) (8.5)	3.6 (3.5)	1.5 (0.7)
Week 112	4 (7.0) (11.4)	2.3 (1.8)	1.1 (0.6)	11 (20.4) (30.6)	5.0 (12.8)	1.2 (1.0)	3 (4.8) (7.0)	2.7 (3.3)	1.1 (0.5)
Week 160	2 (3.5) (6.7)	2.2 (2.1)	1.1 (0.6)	11 (20.4) (36.7)	3.2 (5.1)	1.1 (0.8)	7 (11.3) (17.5)	2.1 (1.8)	1.0 (0.5)
Week 208	4 (7.0) (14.3)	1.5 (1.2)	1.1 (0.6)	7 (13.0) (25.9)	3.1 (5.9)	1.3 (0.9)	2 (3.2) (5.7)	2.1 (1.5)	1.0 (0.5)
Week 256	6 (10.5) (30.0)	1.4 (1.4)	0.9 (0.4)	2 (3.7) (12.5)	4.0 (7.5)	1.4 (1.0)	3 (4.8) (16.7)	1.6 (1.3)	0.8 (0.3)
Week 292	1 (1.8) (16.7)	1.1 (0.6)	1.0 (0.5)	2 (3.7) (50.0)	0.6 (0.6)	0.6 (0.1)	1 (1.6) (20.0)	1.6 (0.9)	1.1 (0.1)

*Duration of pasireotide exposure in the crossover group was 24 weeks shorter than the ‘study visit’, as these patients did not receive pasireotide during the 24-week core phase; ^†^Baseline values are shown at core study baseline for the pasireotide 40 mg and 60 mg groups, and at extension baseline for the crossover group; ^‡^Response rate was calculated (i) using the ITT principle for all patients who received pasireotide and (ii) for patients who reached the scheduled visit.

ITT, intention to treat; pts, patients; sIGF-I, standardized IGF-I (IGF-I/ULN).

Sixty-four patients (37.0%) achieved GH <1.0 µg/L and normal IGF-I at some point during the core or extension phase. Of these patients, 13 (20.3%) achieved a first response within 3 months of treatment initiation, nine (14.1%) did so after 3–6 months of treatment, and 42 (65.6%) after at least 6 months of treatment (Table 5). For patients with a first response after 6 months, the median time to response in the pasireotide 40 mg, 60 mg, and crossover groups, respectively, was 20.3 (*n* = 15), 18.4 (*n* = 15), and 26.8 (*n* = 12) months (range: 9.2–58.9 months). Patient characteristics at baseline were similar irrespective of time of first response (Table 5).

**Table 5:** Baseline characteristics of early and late responders.Response defined as the first occurrence of a reduction of mean GH to <1.0 µg/L and normalization of IGF-I after initiation of pasireotide in the core or extension phase. Data are presented as *n* (%) unless indicated otherwise.

First response	Long-acting pasireotide 40 mg, *n* = 57			Long-acting pasireotide 60 mg, *n* = 54			Crossover to pasireotide, *n* = 62		
	≤3 months	>3–6 months	>6 months	≤3 months	>3–6 months	>6 months	≤3 months	>3–6 months	>6 months
*n*	3	2	15	7	4	15	3	3	12
Age, years*	50.3 (4.9)	50.5 (2.1)	45.9 (14.74)	55.0 (17.0)	39.3 (11.3)	45.9 (9.15)	53.3 (8.39)	43.7 (18.0)	42.2 (14.3)
Baseline GH, µg/L*	9.2 (7.0)	5.2 (3.4)	5.0 (2.9)	6.8 (3.6)	5.1 (2.7)	4.1 (2.0)	4.6 (1.0)	4.8 (2.3)	4.9 (1.7)
Baseline IGF-I*	390.1 (45.8)	442.2 (228.0)	592.8 (133.6)	658.8 (233.5)	477.8 (108.1)	626.7 (155.4)	645.3 (72.8)	633.7 (174.0)	592.4 (161.4)
Sex, *n* (%)									
Male	2 (66.7)	1 (50.0)	7 (46.7)	3 (42.9)	2 (50.0)	6 (40.0)	3 (100.0)	2 (66.7)	6 (50.0)
Female	1 (33.3)	1 (50.0)	8 (53.3)	4 (57.1)	2 (50.0)	9 (60.0)	0 (0.0)	1 (33.3)	6 (50.0)
Tumour volume category at baseline, *n* (%)									
Microadenoma^†^	2 (66.7)	2 (100.0)	11 (73.3)	4 (57.1)	4 (100.0)	7 (46.7)	1 (33.3)	1 (33.3)	11 (91.7)
Macroadenoma^‡^	0 (0.0)	0 (0.0)	1 (6.7)	0 (0.0)	0 (0.0)	1 (6.7)	0 (0.0)	0 (0.0)	0 (0.0)
Missing	1 (33.3)	0 (0.0)	3 (20.0)	3 (42.9)	0 (0.0)	7 (46.7)	2 (66.7)	2 (66.7)	1 (8.3)

*Data are presented as mean (S.D.); ^†^corresponding to a tumour diameter of ≤10 mm; ^‡^corresponding to a tumour diameter of >10 mm.

### Dose titration

In patients randomized to pasireotide 40 mg and for those in the crossover group who started with pasireotide 40 mg in the extension, 68.4% (39/57) and 59.7% (37/62), respectively, received a dose increase to 60 mg during the extension phase; 28.2% (11/39) and 21.6% (8/37) of these patients, respectively, achieved GH <1.0 µg/L and normal IGF-I. For patients randomized to pasireotide 60 mg, 9.3% (5/54) had a dose decrease to 40 mg because of AEs.

### Clinical symptoms of acromegaly

**Figure 1:**
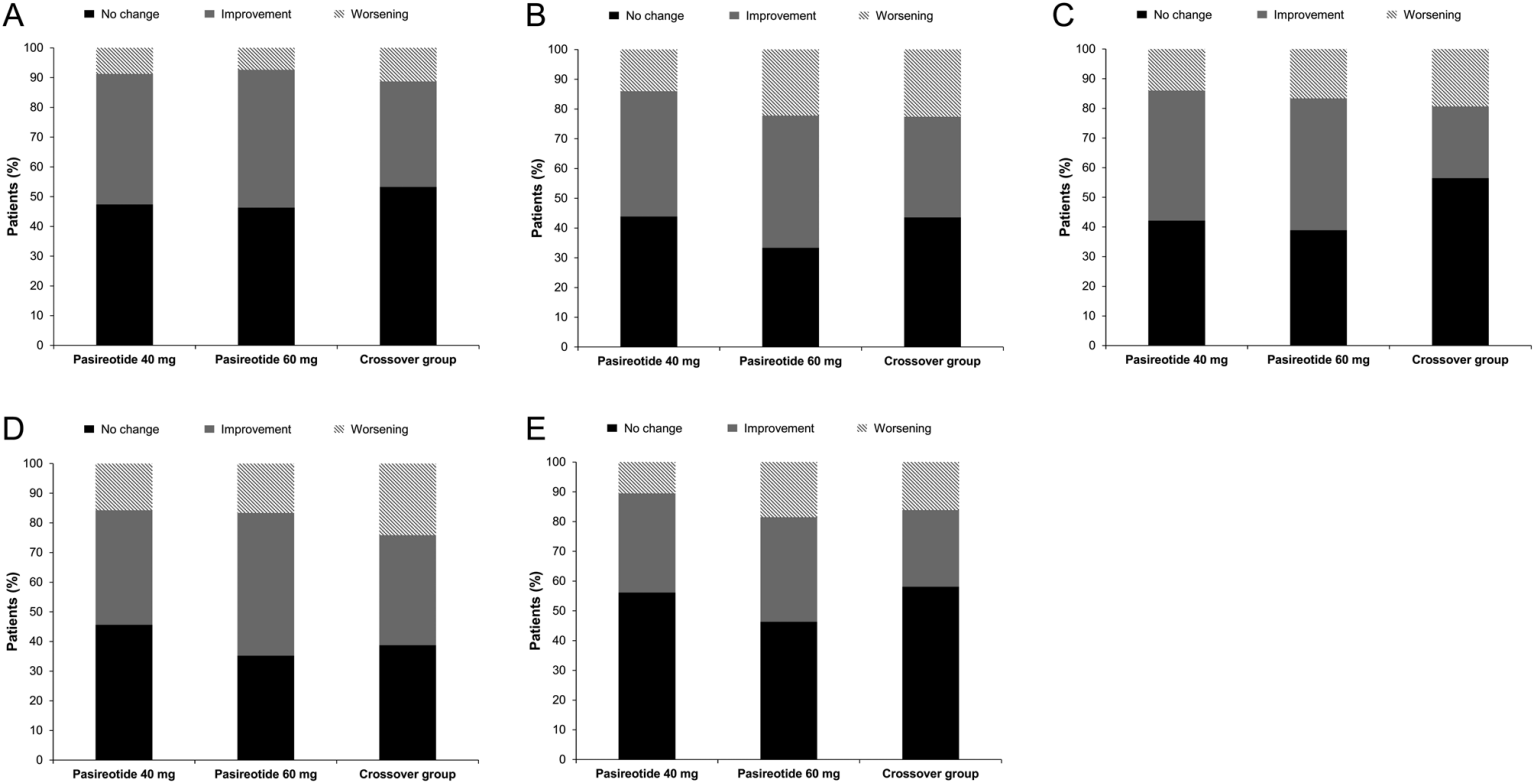
No change, improvements or worsening in key symptoms of acromegaly: (A) headache; (B) fatigue; (C) perspiration; (D) osteoarthralgia; (E) paraesthesia.

Improvements in key symptoms of acromegaly (headache, fatigue, perspiration, paraesthesia and osteoarthralgia) were observed in all treatment groups (Fig. 1). Symptoms emerged in a minority of patients in whom no symptoms were present at baseline (headache, *n* = 9/68; fatigue, *n* = 14/48; perspiration, *n* = 17/75; osteoarthralgia, *n* = 16/56; paraesthesia, *n* = 13/94), but these were generally mild to moderate in severity (Fig. 1).

### Safety and tolerability of long-acting pasireotide

For patients randomized to pasireotide 40 mg and 60 mg, 61/63 (96.8%) and 60/62 (96.8%), as well as 61/62 (98.4%) patients in the crossover group, experienced at least one AE. The most common AEs regardless of study drug relationship were hyperglycaemia (39.7, 40.3, and 25.8%, respectively), diabetes mellitus (31.7, 40.3, and 29.0%, respectively), and cholelithiasis (34.9, 33.9, and 30.6%, respectively; Table 6). AEs resulted in 18.5% (32/173) of patients discontinuing treatment (9 patients each in the crossover group and those randomized to pasireotide 40 mg, and 14 patients randomized to pasireotide 60 mg); of the patients in the pasireotide 40 mg and 60 mg arms, respectively, who stopped pasireotide treatment because of AEs, four and five discontinued during the core phase. Drug-related serious AEs were reported in 5 (7.9%) patients randomized to pasireotide 40 mg (anaemia (*n* = 1), abdominal pain (*n* = 1), cholecystitis (*n* = 1), cholelithiasis (*n* = 2), increased blood glucose (*n* = 1), hyperglycaemia (*n* = 1), diabetic metabolic decompensation (*n* = 1), deep vein thrombosis (*n* = 1)), 6 (9.7%) patients randomized to pasireotide 60 mg (bile duct stone (*n* = 1), cholecystitis (*n* = 1), cholelithiasis (*n* = 1), hyperglycaemia (*n* = 1), hypoglycaemia unawareness (*n* = 1), benign pituitary tumour (*n* = 1), suicide attempt (*n* = 1)), and 8 (12.9%) patients in the crossover group (vertigo (*n* = 1), nausea (*n* = 1), acute cholecystitis (*n* = 1), cholecystitis (*n* = 1), cholelithiasis (*n* = 1), liver abscess (*n* = 1), diabetes mellitus (*n* = 2), hyperglycaemia (*n* = 1), dizziness (*n* = 1)).

**Table 6:** Most common adverse events (>10% in any group) regardless of study drug relationship. Data are presented as *n* (%).

Adverse events	Long-acting pasireotide 40 mg*, *n* = 63		Long-acting pasireotide 60 mg*, *n* = 62		Crossover to pasireotide^†, *n* = 62^	
	All grades	Grade 3/4	All grades	Grade 3/4	All grades	Grade 3/4
Total	61 (96.8)	32 (50.8)	60 (96.8)	33 (53.2)	61 (98.4)	31 (50.0)
Hyperglycaemia	25 (39.7)	7 (11.1)	25 (40.3)	6 (9.7)	16 (25.8)	2 (3.2)
Cholelithiasis	22 (34.9)	3 (4.8)	21 (33.9)	1 (1.6)	19 (30.6)	3 (4.8)
Diabetes mellitus	20 (31.7)	1 (1.6)	25 (40.3)	6 (9.7)	18 (29.0)	2 (3.2)
Headache	18 (28.6)	0	6 (9.7)	3 (4.8)	8 (12.9)	0
Diarrhoea	14 (22.2)	0	17 (27.4)	1 (1.6)	11 (17.7)	1 (1.6)
Back pain	13 (20.6)	1 (1.6)	7 (11.3)	1 (1.6)	3 (4.8)	1 (1.6)
Abdominal pain	10 (15.9)	1 (1.6)	10 (16.1)	0	6 (9.7)	0
Anaemia	10 (15.9)	1 (1.6)	10 (16.1)	2 (3.2)	16 (25.8)	0
Hypoglycaemia	7 (11.1)	0	7 (11.3)	2 (3.2)	4 (6.5)	0
Dizziness	8 (12.7)	0	3 (4.8)	0	3 (4.8)	1 (1.6)
Pyrexia	8 (12.7)	0	1 (1.6)	0	2 (3.2)	0
Influenza	9 (14.3)	0	9 (14.5)	0	5 (8.1)	0
Viral upper RTI	7 (11.1)	0	9 (14.5)	0	5 (8.1)	0
Hypertension	7 (11.1)	1 (1.6)	4 (6.5)	1 (1.6)	5 (8.1)	2 (3.2)
Haematuria	7 (11.1)	0	2 (3.2)	0	0	0
Vomiting	8 (12.7)	0	1 (1.6)	0	0	0
Nausea	7 (11.1)	0	7 (11.3)	0	3 (4.8)	1 (1.6)
Arthralgia	7 (11.1)	0	9 (14.5)	3 (4.8)	3 (4.8)	0
Urinary tract infection	6 (9.5)	0	5 (8.1)	0	9 (14.5)	0
Hypercholesterolaemia	5 (7.9)	0	6 (9.7)	0	8 (12.9)	0
Alopecia	3 (4.8)	0	8 (12.9)	0	0	0

Hyperglycaemia was defined as a post-baseline FPG measurement of ≥126 mg/dL; RTI, respiratory tract infection.

*From start of study drug to study end; ^†^from time of crossover to study end.

In total, five patients were hospitalized for events related to hyperglycaemia: two for events of worsening diabetes mellitus, one of which led to treatment discontinuation and was ongoing at last assessment, with the other resolving without requiring treatment discontinuation; two for hyperglycaemia, both of which resolved without requiring treatment cessation; and one for diabetic decompensation (grade 4 diabetes), which resolved following treatment discontinuation. There were no instances of diabetic ketoacidosis or hyperglycaemic hyperosmolar state. Changes in values of FPG and HbA_1c_ from baseline to end of the extension phase in the three treatment arms are shown in Table 7.

**Table 7:** Mean FPG and HbA_1c_ values at the end of the extension study and change in actual values in FPG and HbA_1c_ from baseline to end of the extension.

Baseline diabetic status	Long-acting pasireotide 40 mg		Long-acting pasireotide 60 mg		Crossover group	
	Actual	Change from baseline	Actual	Change from baseline	Actual	Change from baseline
FPG, mg/dL						
Diabetic, *n* Mean (s.d.) Median (range)	24 136 (32) 137 (85–189)	24 22 (32) 22 (–43to79)	21 168 (80) 148 (99–364)	21 52 (84) 20 (–40to238)	26 152 (50) 135 (97–303)	26 37 (43) 25 (–9to168)
Pre-diabetic, *n* Mean (s.d.) Median (range)	9 117 (30) 115 (74–159)	9 17 (34) 12 (–36to74)	11 133 (38) 121 (96–227)	11 28 (38) 20 (–2to131)	10 116 (18) 112 (92–142)	10 15 (16) 8 (–4to44)
Normal glucose tolerance, *n* Mean (s.d.) Median (range)	2 125 (1) 125 (124–126)	2 28 (4) 28 (25–30)	2 158 (6) 158 (153–162)	2 71 (5) 71 (67–74)	1 96 (–) 96 (–)	1 10 (–) 10 (–)
HbA_1c_, %						
Diabetic, *n* Mean (s.d.) Median (range) Pre-diabetic, *n* Mean (s.d.) Median (range) Normal glucose tolerance, *n* Mean Median (range)	23 7.0 (1.1) 6.8 (5.6–9.5) 9 6.1 (0.6) 6.1 (5.1–7.0) 2 5.9 (0.1) 5.8 (5.8–5.9)	23 0.8 (0.9) 1.0 (–1.5to2.3) 9 0.5 (0.5) 0.4 (–0.1to1.3) 2 0.3 (0.1) 0.3 (0.2–0.3)	20 7.9 (1.9) 7.2 (5.8–12.2) 11 6.7 (2.1) 6.0 (5.6–13.0) 3 6.8 (1.0) 6.5 (6.0–7.9)	20 1.8 (1.9) 1.0 (–0.3to6.1) 11 1.0 (2.1) 0.3 (–0.2to7.1) 3 1.4 (0.9) 1.2 (0.6–2.4)	29 7.3 (1.1) 7.4 (5.3–10.1) 10 6.1 (0.5) 6.2 (5.4–6.9) 1 6.2 (–) 6.2 (–)	29 1.3 (1.0) 1.3 (–0.1to4.0) 10 0.4 (0.4) 0.4 (–0.3to0.9) 1 0.6 (–) 0.6 (–)

There were two deaths during the extension phase, both in the pasireotide 40 mg group: one patient died because of a brain oedema that was not suspected to be related to treatment, and one patient discontinued study treatment because of an AE of lung cancer that ultimately led to death.

### Hyperglycaemia management

Across the three treatment groups, 75.0–100.0% of patients who were pre-diabetic or had normal glucose tolerance at baseline, and 31.3–55.3% of patients who were diabetic at baseline, had a last available HbA_1c_ value of <7% (<53.0 mmol/mol) at study end (Table 8).

**Table 8:** Number of patients with last available HbA_1c_ <7%* at study end, according to baseline diabetic status. Data based on 173 patients who received at least one dose of pasireotide during the extension phase. See Methods section for definitions of diabetic status.

Baseline diabetic status	Long-acting pasireotide 40 mg	Long-acting pasireotide 60 mg	Crossover group
Diabetic, *n* HbA_1c_, *n* (%)	38 21 (55.3)	32 10 (31.3)	44 22 (50.0)
Pre-diabetic, *n* HbA_1c_, *n* (%)	12 11 (91.7)	12 9 (75.0)	17 16 (94.1)
NGT, *n* HbA_1c_, *n* (%)	7 6 (85.7)	10 9 (90.0)	1 1 (100.0)

NGT, normal glucose tolerance.

*Target HbA_1c_ level set by the American Diabetes Association and European Association for the Study of Diabetes (11, 12).

During the core and extension phase, the use of concomitant antidiabetic medication was similar across the three treatment arms (pasireotide 40 mg, *n* = 42 (66.7%); pasireotide 60 mg, *n* = 37 (59.7%); crossover (extension only), *n* = 41 (66.1%)). In the pasireotide 40 mg, pasireotide 60 mg and crossover groups, respectively, 47.6, 46.8 and 50.0% received a form of metformin, 23.8, 19.4 and 25.8% received a dipeptidyl peptidase 4 inhibitor, 27.0, 24.2 and 25.8% received a sulfonylurea, and 9.5, 8.1 and 8.1% received insulin and analogues.

Among diabetic patients randomized to either dose of pasireotide or who received pasireotide in the crossover group, use of antidiabetic medication was less frequent after the start of study relative to baseline (Table 9). However, an increase in use of antidiabetic medication after the start of study versus baseline was observed for patients randomized to either dose of pasireotide and who were pre-diabetic or had normoglycaemia at baseline. Pre-diabetic patients in the crossover group also had increased use of antidiabetic medication after the start of the extension phase relative to extension baseline.

**Table 9:** Number of patients who had antidiabetic medication at baseline or after start of study, according to baseline diabetic status. Data based on 173 patients who received at least one dose of pasireotide during the extension phase. See Methods section for definitions of diabetic status.

Baseline diabetic status	Long-acting pasireotide 40 mg		Long-acting pasireotide 60 mg		Crossover group	
	At baseline	After start of study	At baseline	After start of study	At extension baseline	After start of extension
Diabetic, *n* ADM, *n* (%)	43 19 (44.2)	43 16 (37.2)	37 17 (45.9)	37 14 (37.8)	44 22 (50.0)	44 15 (34.1)
Pre-diabetic, *n* ADM, *n* (%)	12 0 (0.0)	12 4 (33.3)	13 0 (0.0)	13 4 (30.8)	17 2 (11.8)	17 6 (35.3)
NGT, *n* ADM, *n* (%)	8 0 (0.0)	8 4 (50.0)	12 0 (0.0)	12 3 (25.0)	1 0 (0.0)	1 0 (0.0)

ADM, antidiabetic medication; NGT, normal glucose tolerance.

## Discussion

Uncontrolled or untreated acromegaly has serious physical (2) and psychological health consequences (12), placing patients at higher risk of death than the general population (13, 14), as well as causing multiple comorbid conditions that lead to impaired quality of life (15). Achieving control of GH and IGF-I is a crucial goal of disease management. Normalizing GH and IGF-I can restore the mortality rate in patients with acromegaly to that of the general population (16) and reduce associated comorbidities (17). Regular monitoring of GH and IGF-I levels and proactive changes to treatment if control is not achieved are important approaches to long-term patient management that support the goal of biochemical control.

Data from the prospective, international PAOLA study provided an opportunity to evaluate the prevalence of comorbidities in a large population of patients with difficult-to-treat acromegaly; eligible patients had uncontrolled acromegaly despite ≥6 months of treatment with a first-generation somatostatin analogue. Diabetes was reported in 67% of patients enrolled in the study, which is higher than that previously reported for patients with acromegaly (19–56%) (2). Consistent with previous reports in patients with acromegaly (18), a high proportion of patients enrolled in our study had hypertension (40%) at baseline, while over one-quarter of patients had one or more lipid-related disorders. Approximately half of patients had three or more comorbid disorders at study entry, indicating a high burden of illness in the patient population.

Although first-generation somatostatin analogues have been effective for many years in the clinic, with biochemical control reported in approximately 55% of patients (7), a substantial number of patients remain uncontrolled and are therefore exposed to the health risks associated with excess GH and IGF-I. All enrolled patients were uncontrolled, according to assessments of both GH and IGF-I levels, despite prior treatment with a first-generation somatostatin analogue. Although the reasons for inadequate control with first-generation somatostatin analogues are multifactorial (19, 20), a likely cause is low expression levels of somatostatin receptor subtype (SSTR) 2 on the surface of somatotropinoma cells (21). SSTR5 is also expressed in abundance on somatotropinomas (22), providing rationale for the use of pasireotide in patients uncontrolled on SSTR2-preferential first-generation somatostatin analogues.

In the present study, pasireotide treatment effectively and consistently suppressed GH and IGF-I in a difficult-to-treat patient population for up to 5.8 years of treatment. Notably, of the patients who achieved biochemical control (defined by current recommendations as GH <1.0 µg/L and normal IGF-I) at least once during the core or extension phase, 65.6% first responded after more than 6 months of treatment. Over one-quarter (28%) of patients who received a dose increase of long-acting pasireotide from 40 to 60 mg during the extension were subsequently able to achieve biochemical control. Thus, increasing the dose of pasireotide in patients who have not achieved biochemical control allows more patients to achieve control of GH and IGF-I levels.

Improvements in key acromegaly-associated symptoms were also observed with pasireotide treatment, regardless of baseline symptom severity. This finding is clinically significant given that patients were burdened with a multiplicity of symptoms at study baseline despite prior treatment at the start of the study.

The long-term safety profile of pasireotide was similar to that observed during the 6-month core study; hyperglycaemia, diabetes mellitus, and cholelithiasis were the most common AEs. There were no new safety signals identified in the extension phase compared with the core phase. Importantly, only 13.3% of patients who received pasireotide during the extension discontinued treatment because of AEs. As pasireotide was used for several years during the study, the low rate of AE-related discontinuations indicates that hyperglycaemia, the most common side effect with pasireotide, is manageable. This finding is of particular clinical significance given that 67.2% of patients had diabetes mellitus at core baseline, although it should be noted that there are differences in the glycaemic parameters used to define diabetes and pre-diabetes in our study compared with the current guidelines from the American Diabetes Association (23). Furthermore, it is possible that some patients could have been incorrectly classified as diabetic in our study based on their use of antidiabetic medications, such as metformin, which is frequently prescribed for patients with insulin resistance without a diagnosis of diabetes. The prolonged exposure to pasireotide during the course of the study may also account for the rate of drug-related serious AEs observed, while the low discontinuation rate in the study in spite of this likely reflects the significant burden of uncontrolled acromegaly; patients eligible for this extension study had uncontrolled acromegaly while receiving maximal-dose octreotide or lanreotide for at least 12 months.

As previously mentioned, diabetes mellitus is one of the most common comorbidities to occur in patients with acromegaly and exposes patients to increased risk of mortality. Management of diabetes and other comorbidities is an important therapeutic goal in the treatment of acromegaly (3); while treatment with first-generation somatostatin analogues typically increases FPG levels during the first month of therapy, attainment of disease control (defined in this instance as mean fasting GH ≤2.5 µg/L and normal age- and sex-matched IGF-I) after 12 months of treatment is strongly associated with maintenance of glucose levels or even a reduction in some patients (24). Conversely, glucose levels tend to increase in most pasireotide-treated patients. Pasireotide-associated hyperglycaemia typically occurs within the first month of treatment (25) and is manageable with antidiabetic medication in most instances or reversible upon discontinuation of pasireotide if uncontrolled with antidiabetic treatment. In our study, most patients had an HbA_1c_ level below the target set by the American Diabetes Association (<7%) at their last assessment, demonstrating that they were not exposed to prolonged periods of hyperglycaemia. Vigilant monitoring of glucose levels during pasireotide treatment is required, with prompt action taken as necessary, starting with the initiation of antidiabetic medication.

Pegvisomant, a GH-receptor antagonist, is an alternative therapeutic option for patients with impaired glucose tolerance (26). Although pegvisomant has been shown to control IGF-I levels in at least 63.2% of patients after 5 years of treatment and improve glucose tolerance, its mechanism of action results in significantly increased GH levels (27). Additionally, pegvisomant therapy requires monitoring of hepatic function and evaluation of tumour size, although tumour progression is rare. Combination therapy also presents a viable therapeutic alternative, which is increasingly employed in patients with uncontrolled acromegaly. Treatment with various combinations of cabergoline, octreotide/lanreotide and pegvisomant, and more recently pasireotide and pegvisomant, have been shown to be efficacious in subsets of patients with acromegaly (28, 29, 30).

Given the chronic nature of acromegaly, prolonged administration of medical therapy is often necessary to maintain long-term suppression of GH and IGF-I levels. In the current study, pasireotide demonstrated long-term sustained suppression of both GH and IGF-I levels, with improvements in key symptoms and a low AE-related discontinuation rate suggestive of acceptable tolerability. At present, pasireotide is indicated in the EU for adult patients with acromegaly for whom surgery has failed or is not an option and who are inadequately controlled on other first-generation somatostatin analogues. In the United States, pasireotide can be given as a first-line medical therapy in patients for whom surgery has failed or is not an option.

With several effective options available for the treatment of acromegaly, identifying the right treatment for the right patient is critical. In addition to key prospective and real-world studies providing efficacy and safety data, cost-effectiveness analyses are also valuable in facilitating clinical decision making. One study reviewed current cost-effectiveness analyses of acromegaly treatments, highlighting several key limitations with these studies (31). As such, there remains a need for additional, robust cost-effectiveness analyses to be conducted for all medical treatment options for acromegaly.

In the core phase of this study, patients were randomized to receive double-blind pasireotide 40 or 60 mg, or continued treatment with open-label octreotide/lanreotide. The unavoidable difference in blinding between pasireotide and octreotide/lanreotide recipients is a limitation of this study.

In conclusion, long-acting pasireotide demonstrated a positive benefit/risk profile for up to 5.8 years of treatment in patients with acromegaly uncontrolled on prior first-generation somatostatin analogue therapy who had a high prevalence of baseline comorbidity, including glucose-related disorders.

## Supplementary Material

Pasireotide for acromegaly: long-term outcomes from an extension to the Phase III (PAOLA) study

Supplementary Figure 1. Patient flow

Supplementary Figure 2. Number (%) of patients from the randomized population (N=198) according to core baseline comorbidity group or combinations thereof

## Declaration of interest

A C has received consultancy and speaker fees from Novartis, Pfizer and Ipsen. M D B has served on steering committees for Chiasma, Ipsen and Novartis and has received speaker fees from Ipsen and Novartis, as well as clinical research grants from Ipsen, Novartis and Pfizer. T B has received institutional research support from Pfizer and consultancy/lectureship fees from Novartis, Ipsen, Strongbridge and Pfizer. M F is the principal investigator for research grants to Oregon Health & Science University received from Chiasma, Crinetics, Ionis, Novartis and Pfizer and an *ad hoc* scientific consultant to Chiasma, Crinetics, Ionis, Ipsen, Novartis and Pfizer. M G has served as a principal investigator in clinical trials conducted by Novartis. G R has received institutional research support from Novartis and Ipsen and consultancy and lectureship fees from Novartis, Ipsen, Chiasma and Pfizer. I S has received research grants and consultancy and lectureship fees from Novartis, Chiasma and Pfizer. M R G has received research grants and speaker fees from Novartis, Ipsen and Pfizer, has attended advisory boards for Novartis and Ionis, and has been a principal investigator in clinical trials conducted by Novartis and Ipsen. J F, P G and A M P are employees of Novartis.

## Funding

This study was funded by Novartis Pharma AG. Financial support for medical editorial assistance was provided by Novartis Pharmaceuticals Corporation.

## Data sharing

Novartis is committed to sharing with qualified external researchers access to patient-level data and supporting clinical documents from eligible studies. These requests are reviewed and approved by an independent review panel on the basis of scientific merit. All data provided are anonymized to respect the privacy of patients who have participated in the trial, in line with applicable laws and regulations. This trial data availability is in accordance with the criteria and process described on www.clinicalstudydatarequest.com.

## References

[1] Sanno N Teramoto A Osamura RY Horvath E Kovacs K Lloyd RV Scheithauer BW Pathology of pituitary tumors. Neurosurgery Clinics of North America 2003 14 25–39, vi. (10.1016/s1042-3680(02)00035-9) 12690977

[2] Colao A Ferone D Marzullo P Lombardi G Systemic complications of acromegaly: epidemiology, pathogenesis, and management. Endocrine Reviews 2004 25 102–152. (10.1210/er.2002-0022) 14769829

[3] Katznelson L Laws ER Jr Melmed S Molitch ME Murad MH Utz A Wass JA & Endocrine Society. Acromegaly: an Endocrine Society clinical practice guideline. Journal of Clinical Endocrinology and Metabolism 2014 99 3933–3951. (10.1210/jc.2014-2700) 25356808

[4] Colao A Vandeva S Pivonello R Grasso LF Nachev E Auriemma RS Kalinov K Zacharieva S Could different treatment approaches in acromegaly influence life expectancy? A comparative study between Bulgaria and Campania (Italy). European Journal of Endocrinology 2014 171 263–273. (10.1530/EJE-13-1022) 24878680

[5] Gadelha MR Kasuki L Lim DST Fleseriu M Systemic complications of acromegaly and the impact of the current treatment landscape: an update. Endocrine Reviews 2019 40 268–332. (10.1210/er.2018-00115) 30184064

[6] Oberg K Lamberts SW Somatostatin analogues in acromegaly and gastroenteropancreatic neuroendocrine tumours: past, present and future. Endocrine-Related Cancer 2016 23 R551–R566. (10.1530/ERC-16-0151) 27697899

[7] Carmichael JD Bonert VS Nuño M Ly D Melmed S Acromegaly clinical trial methodology impact on reported biochemical efficacy rates of somatostatin receptor ligand treatments – a meta-analysis. Journal of Clinical Endocrinology and Metabolism 2014 99 1825–1833. (10.1210/jc.2013-3757) 24606084 PMC4010703

[8] Colao A Auriemma RS Pivonello R Kasuki L Gadelha MR Interpreting biochemical control response rates with first-generation somatostatin analogues in acromegaly. Pituitary 2016 19 235–247. (10.1007/s11102-015-0684-z) 26519143 PMC4858561

[9] Colao A Bronstein MD Freda P Gu F Shen CC Gadelha M Fleseriu M van der Lely AJ Farrall AJ Hermosillo Reséndiz K ***et al*** Pasireotide versus octreotide in acromegaly: a head-to-head superiority study. Journal of Clinical Endocrinology and Metabolism 2014 99 791–799. (10.1210/jc.2013-2480) 24423324 PMC3965714

[10] Gadelha MR Bronstein MD Brue T Coculescu M Fleseriu M Guitelman M Pronin V Raverot G Shimon I Lievre KK ***et al*** Pasireotide versus continued treatment with octreotide or lanreotide in patients with inadequately controlled acromegaly (PAOLA): a randomised, phase 3 trial. Lancet: Diabetes and Endocrinology 2014 2 875–884. (10.1016/S2213-8587(14)70169-X) 25260838

[11] Giustina A Barkan A Casanueva FF Cavagnini F Frohman L Ho K Veldhuis J Wass J von Werder K Melmed S Criteria for cure of acromegaly: a consensus statement. Journal of Clinical Endocrinology and Metabolism 2000 85 526–529. (10.1210/jcem.85.2.6363) 10690849

[12] Leon-Carrion J Martin-Rodriguez JF Madrazo-Atutxa A Soto-Moreno A Venegas-Moreno E Torres-Vela E Benito-López P Galvez MA Tinahones FJ Leal-Cerro A Evidence of cognitive and neurophysiological impairment in patients with untreated naive acromegaly. Journal of Clinical Endocrinology and Metabolism 2010 95 4367–4379. (10.1210/jc.2010-0394) 20554710

[13] Holdaway IM Bolland MJ Gamble GD A meta-analysis of the effect of lowering serum levels of GH and IGF-I on mortality in acromegaly. European Journal of Endocrinology 2008 159 89–95. (10.1530/EJE-08-0267) 18524797

[14] Sherlock M Ayuk J Tomlinson JW Toogood AA Ragon-Alonso A Sheppard MC Bates AS Stewart PM Mortality in patients with pituitary disease. Endocrine Reviews 2010 31 301–342. (10.1210/er.2009-0033) 20086217

[15] T’Sjoen G Bex M Maiter D Velkeniers B Abs R Health-related quality of life in acromegalic subjects: data from AcroBel, the Belgian registry on acromegaly. European Journal of Endocrinology 2007 157 411–417. (10.1530/EJE-07-0356) 17893254

[16] Holdaway IM Rajasoorya RC Gamble GD Factors influencing mortality in acromegaly. Journal of Clinical Endocrinology and Metabolism 2004 89 667–674. (10.1210/jc.2003-031199) 14764779

[17] Varadhan L Reulen RC Brown M Clayton RN The role of cumulative growth hormone exposure in determining mortality and morbidity in acromegaly: a single centre study. Pituitary 2016 19 251–261. (10.1007/s11102-015-0700-3) 26724807

[18] Bondanelli M Ambrosio MR degli Uberti EC Pathogenesis and prevalence of hypertension in acromegaly. Pituitary 2001 4 239–249. (10.1023/a:1020798430884) 12501974

[19] Colao A Auriemma RS Lombardi G Pivonello R Resistance to somatostatin analogs in acromegaly. Endocrine Reviews 2011 32 247–271. (10.1210/er.2010-0002) 21123741

[20] Gadelha MR Kasuki L Korbonits M Novel pathway for somatostatin analogs in patients with acromegaly. Trends in Endocrinology and Metabolism 2013 24 238–246. (10.1016/j.tem.2012.11.007) 23270713

[21] Wildemberg LE Neto LV Costa DF Nasciuti LE Takiya CM Alves LM Rebora A Minuto F Ferone D Gadelha MR Low somatostatin receptor subtype 2, but not dopamine receptor subtype 2 expression predicts the lack of biochemical response of somatotropinomas to treatment with somatostatin analogs. Journal of Endocrinological Investigation 2013 36 38–43. (10.3275/8305) 22472799

[22] Chinezu L Vasiljevic A Jouanneau E Francois P Borda A Trouillas J Raverot G Expression of somatostatin receptors, SSTR2A and SSTR5, in 108 endocrine pituitary tumors using immunohistochemical detection with new specific monoclonal antibodies. Human Pathology 2014 45 71–77. (10.1016/j.humpath.2013.08.007) 24182563

[23] American Diabetes Association. 2. Classification and diagnosis of diabetes: standards of medical care in Diabetes-2019. Diabetes Care 2019 42 S13–S28. (10.2337/dc19-S002) 30559228

[24] Colao A Auriemma RS Savastano S Galdiero M Grasso LF Lombardi G Pivonello R Glucose tolerance and somatostatin analogues treatment in acromegaly: a 12-month study. Journal of Clinical Endocrinology and Metabolism 2009 94 2907–2914. (10.1210/jc.2008-2627) 19491229

[25] Silverstein JM Hyperglycemia induced by pasireotide in patients with Cushing’s disease or acromegaly. Pituitary 2016 19 536–543. (10.1007/s11102-016-0734-1) 27405306 PMC4996868

[26] Melmed S Bronstein MD Chanson P Klibanski A Casanueva FF Wass JAH Strasburger CJ Luger A Clemmons DR Giustina A A Consensus Statement on acromegaly therapeutic outcomes. Nature Reviews: Endocrinology 2018 14 552–561. (10.1038/s41574-018-0058-5) PMC7136157 30050156

[27] van der Lely AJ Hutson RK Trainer PJ Besser GM Barkan AL Katznelson L Klibanski A Herman-Bonert V Melmed S Vance ML ***et al*** Long-term treatment of acromegaly with pegvisomant, a growth hormone receptor antagonist. Lancet 2001 358 1754–1759. (10.1016/s0140-6736(01)06844-1) 11734231

[28] Lim DS Fleseriu M The role of combination medical therapy in the treatment of acromegaly. Pituitary 2017 20 136–148. (10.1007/s11102-016-0737-y) 27522663

[29] Muhammad A van der Lely AJ Delhanty PJD Dallenga AHG Haitsma IK Janssen JAMJL Neggers SJCMM Efficacy and safety of switching to pasireotide in acromegaly patients controlled with pegvisomant and first-generation somatostatin analogues (PAPE study). Journal of Clinical Endocrinology and Metabolism 2018 103 586–595. (10.1210/jc.2017-02017) 29155991

[30] Muhammad A Coopmans EC Delhanty PJD Dallenga AHG Haitsma IK Janssen JAMJL van der Lely AJ Neggers SJCMM Efficacy and safety of switching to pasireotide in acromegaly patients controlled with pegvisomant and somatostatin analogues: PAPE extension study. European Journal of Endocrinology 2018 179 269–277. (10.1530/EJE-18-0353) 30076159

[31] Leonart LP Borba HHL Ferreira VL Riveros BS Pontarolo R Cost-effectiveness of acromegaly treatments: a systematic review. Pituitary 2018 21 642–652. (10.1007/s11102-018-0908-0) 30159696

