# Supplementary material for: Pasireotide for acromegaly: long-term outcomes from an extension to the Phase III PAOLA study
Source: Eur J Endocrinol. 2020 Mar 27;182(6):583–94. doi: 10.1530/EJE-19-0762 (PMC7222286; doi:10.1530/EJE-19-0762)
Supplement: Pasireotide for acromegaly: long-term outcomes from an extension to the Phase III (PAOLA) study [file supplementary_table_1.pdf]

Pasireotide for acromegaly: long-term outcomes from an extension to the Phase III (PAOLA) study

Supplementary appendix

List of independent ethics committees and institutional review boards that provided ethics approval for the study

Ethik-Kommission der Ludwig-Maximilians-Universität (Munich, Germany), Friedrich-Alexander-Universität Erlangen-Nürnberg (Erlangen, Germany), Julius-Maximilians-Universität Würzburg (Würzburg, Germany), Commisie medsche ethiek (Leuven, Belgium), Comitê de Ética em Pesquisa em Seres Humanos da Faculdade de Ciencias Medicas-UNICAMP – SP (Campinas, Brazil), Comitê de Ética em Pesquisa da Faculdade de Medicina – USP (São Paulo, Brazil), Comitê de Ética em Pesquisa do Hospital Universitário Walter Cantidio (Fortaleza, Brazil), Comitê de Ética em Pesquisa da Unifesp/EPM (São Paulo, Brazil), Comitê de Ética em Pesquisa do Hospital Municipal Sao Jose (Joinville, Brazil), Comitê de Ética em Pesquisa da Faculdade de Medicina de Botucatu (Botucatu, Brazil), Comite de Etica en Investigacion con seres humanos – Fundacion Universitaria de Ciencias de la Salud (Bogota, Colombia), Comite de Etica en Investigación Centro Medico Imbanaco (Cali, Colombia), Helsinki Committee (Petah Tikva, Israel), Comitato Etico (Genoa, Italy), Comitato Etico (Naples, Italy), Comitato Etico Interaziendale (Turin, Italy), Comitato Etico Dell' Universita Cattolica del Sacro Cuore (Rome, Italy), Comitato Etico Scientifico (Messina, Italy), REK sør-øst (Oslo, Norway), Komisja Bioetyczna przy Akademii Medycznej we Wroclawiu (Wroclav, Poland), Komisja Bioetyczna przy Akademii Medycznej we Wroclawiu (Wroclav, Poland), Komisja Bioetyczna przy Akademii Medycznej we Wroclawiu (Wroclav, Poland), Comite Etica Investigacion Clinica (Alicante, Spain), Ceic Hospital Universidad Vall' De Hebron (Barcelona, Spain), CEIC Hospital Virgen del Rocio (Seville, Spain), Directorate of Ethics Advisory Committee (Ankara, Turkey), CPP Sud Est Mediterrannee II Marseille, (Marseille, France), NHS Southampton, (Bristol, England), Local Ethic Committee of Endocrinologic Scientific Center (Moscow, Russia), Ethic Committee under Association of Medical and Pharmaceutical Universities (Moscow, Russia), Local Ethic Committee of Altay Regional Clinical Hospital (Barnaul, Russia), Local Ethic Committee of Tyumen State Medical Academy (Tyumen, Russia), National Ethics Committee for Clinical Trial on Medicine (Bucharest, Romania), King Faisal Specialist Hospital and Research Centre – Riyadh (Riyadh, Saudi Arabia), King Khlaid National Guard Hospital Jeddah (Jeddah, Saudi Arabia), Comite de Etica en Investigacion de Instituto Medica Especia Lizardo (Buenos Aires, Argentina), Western IRB (Olympia, WA, USA), Oregon Health & Science University (Portland, OR, USA), Medical School IRB (Ann Arbor, MI, USA), Southwestern Medical Center (Dallas, TX, USA), Comité d'éthique de la recherche sur I'humain – CHUS (Sherbrooke, Canada)

Investigator-defined comorbidity group classifications

Glucose-related disorders were defined as increased blood glucose, diabetes mellitus, impaired fasting glucose, impaired glucose tolerance, hyperglycaemia, and type 2 diabetes mellitus.

Endocrine-related disorders were defined as adrenal insufficiency, secondary adrenocortical insufficiency, androgen deficiency, autoimmune thyroiditis, diabetes insipidus, goitre, gonadotrophin deficiency, hyperparathyroidism, secondary hyperparathyroidism, hyperprolactinaemia, hyperthyroidism, hypogonadism, secondary hypogonadism, hypoparathyroidism, hypopituitarism, hypothyroidism, primary hypothyroidism, secondary hypothyroidism, thyroid cyst, and toxic nodular goitre.

Lipid-related disorders were defined as increased blood triglycerides, dyslipidaemia, hypercholesterolaemia, hyperlipidaemia, hypertriglyceridaemia, and lipid metabolism disorder.

Vascular disorders were defined as hypertension.

All other acromegaly-related disorders not included in the other groups were defined as acrochordon, adenocarcinoma of colon, adrenal adenoma, anxiety, anxiety disorder, aortic valve disease, aortic valve incompetence, arrhythmia, arthralgia, arthropathy, first-degree atrioventricular block, back pain, benign breast neoplasm, bradycardia, breast cancer, left bundle branch block, right bundle branch block, cardiac aneurysm, cardiac hypertrophy, cardiomegaly, cardiomyopathy, carpal tunnel decompression, carpal tunnel syndrome, claustrophobia, colon adenoma, colon cancer, conduction disorder, congestive cardiomyopathy, coronary artery disease, depression, diastolic dysfunction, endometrial cancer, haemangioma of liver, headache, heart valve incompetence, hypertensive cardiomyopathy, hypertensive heart disease, insomnia, intervertebral disc protrusion, intestinal polyp, joint stiffness, large intestine polyp, left ventricular hypertrophy, lipoma, mitral valve incompetence, musculoskeletal pain, myocardial infarction, neck pain, osteoarthritis, osteochondrosis, osteopenia, osteoporosis, panic attack, panic disorder, and ventricular dilatation.

Supplementary Figure 1. Patient flow

Supplementary Figure 2. Number (%) of patients from the randomized population (N=198) according to core baseline comorbidity group or combinations thereof

Data show patient counts associated with all possible combinations of the five comorbidity groups. As a patient could have had only one combination of comorbidities, those combinations in which no patient could be exclusively assigned were excluded. Yellow area represents comorbidities in three or more comorbidity groups
