# Supplementary figures and images for: Pasireotide for acromegaly: long-term outcomes from an extension to the Phase III PAOLA study
Source: Eur J Endocrinol. 2020 Mar 27;182(6):583–94. doi: 10.1530/EJE-19-0762 (PMC7222286; doi:10.1530/EJE-19-0762)

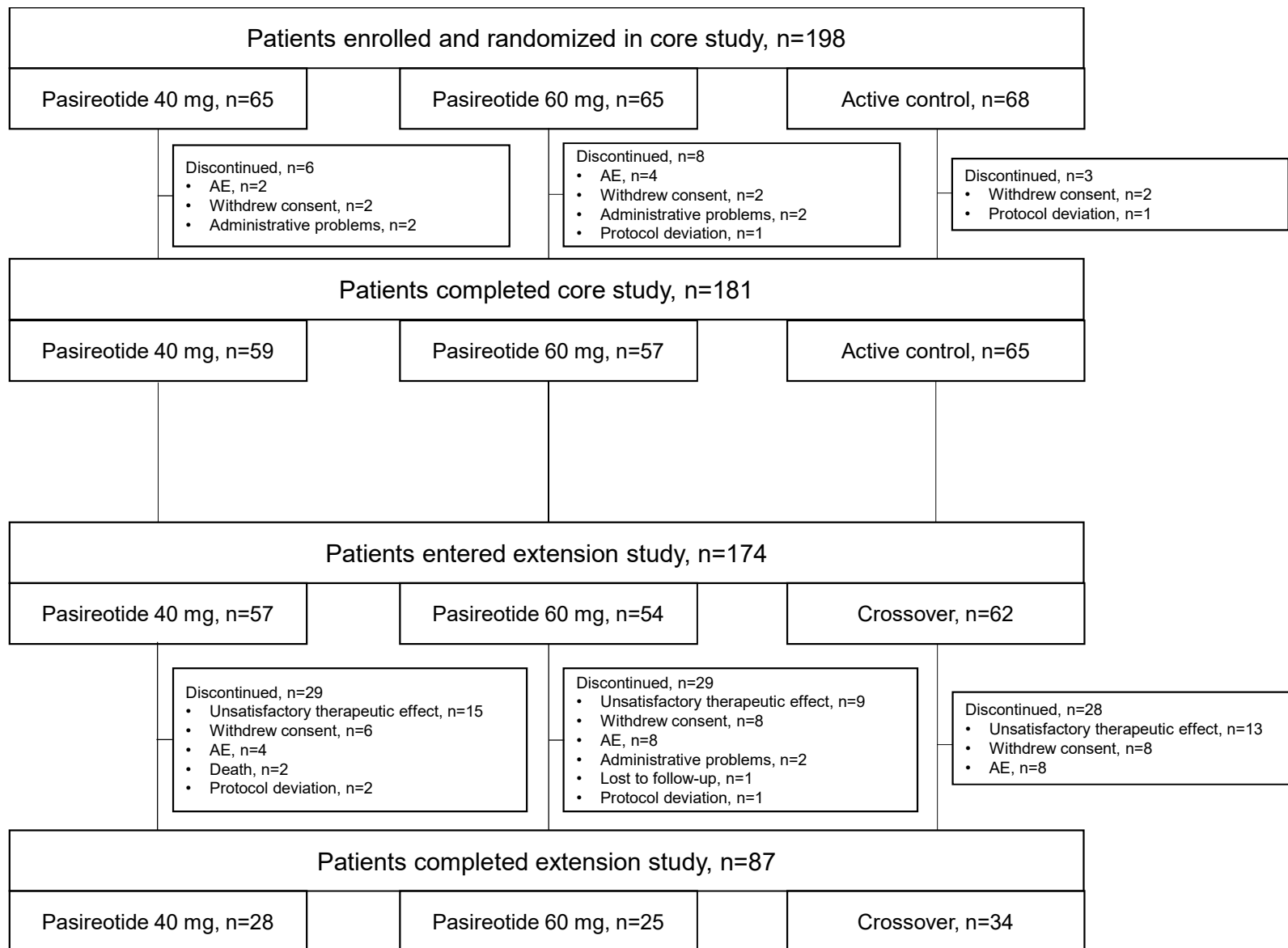

Supplement: Supplementary Figure 1. Patient flow [file supplementary_figure_1.pdf]

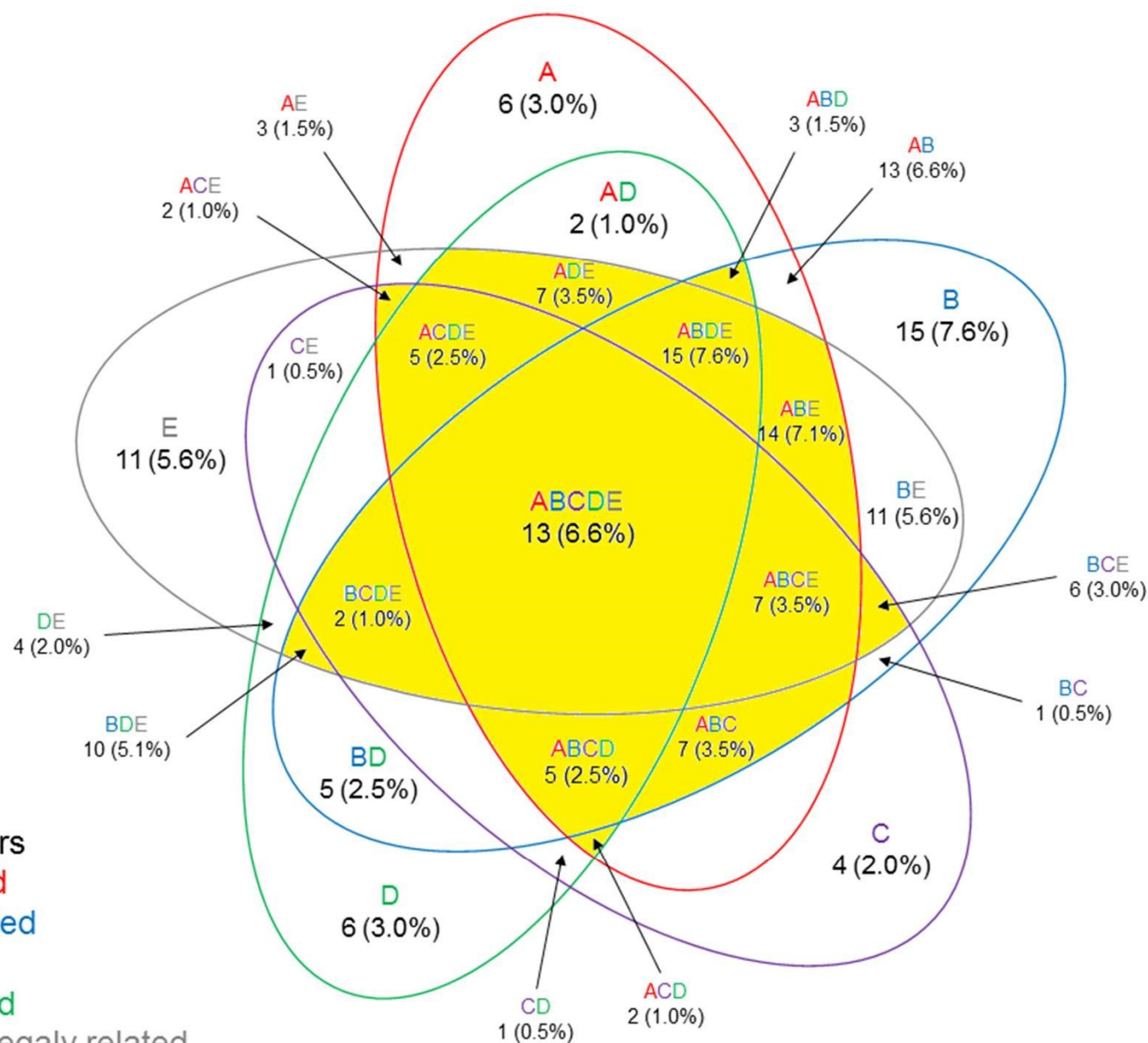

Supplement: Supplementary Figure 2. Number (%) of patients from the randomized population (N=198) according to core baseline comorbidity group or combinations thereof [file supplementary_figure_2.pdf]
